# Great Achievements under Suffering

**Published:** 1894-03-31

**Authors:** 


### Great Achievements Under Suffering.

### V.—LEOPOLD, DUKE OF ALBANY.

The republication in a recent volume of essays of the finely-conceived memoir of the Prince, by Mr. Myers,* should help to keep before men's minds a great example, not only of cheerfulest submission to physical discomfort, but also of the rarer power to turn all pain to high account. So short the life, so lacking in event, so inevitably cut off from action, that many may judge that "achievement" should hardly be reckoned in its compass. But it is good to be reminded sometimes that the ultimate aim of life is less *to do* than to be, and that a noble life, whether passed in the seclusion of a castle or a cottage, can never lack the fruit of high achievement.

The outline of his life is soon told, and indeed is fresh in the memories of all. Born at Buckingham Palace on April 3rd, 1853, his delicate constitution from the first gave rise to the greatest anxiety. His early years were passed in almost incessant ill-health. He could enjoy none of the active pleasures of boyhood, and in spite of the quickness of his intelligence, and his eager delight in books, it was impossible for him to pursue a regular course of education. Mr. Myers' recollections of him at the age of fifteen in Windsor Castle show a nature fretting against restraint, constantly urged by an unconquerable energy to efforts beyond his strength, and doomed to many hours of restless indolence, of idle beating against the bars of his fate. "He was then a most engaging boy, with the physical charm which accompanies the union of high spirit with fragile delicacy, and the moral charm of a nature, whose affections at once vivid and diffident, seemed to beseech the regard and notice which all who knew him were eager to bestow." The inward drama had already begun which lay in "the gradual transformation of his boyish idea of Royal descent as a title to enjoyment, hampered by wearisome restraints, to his manlier view of that high birth as a summons to duty, and his willing submission to its accompanying restrictions as part and parcel of the calling which his whole heart embraced."

In this gradual subjection of all rebellious desires to a high aim the great master was suffering. But there were not wanting many powerful influences to aid in transmuting the bitterness of deprivation to the wholesome medicine of an invigorating restraint. Among the Prince's friends were numbered the wisest and best of the land, and the double portion of home love and sympathy evoked by his isolation was reflected in all those who approached him. "Many visitors to the Castle must remember interviews with the young Prince in his rooms, interviews often prolonged far beyond mere complimentary limits, and leaving behind them the memory of a listener best pleased with what was best worth hearing, and whose transparent face expressed that pleasure with a boy's straightforward charm. There might one meet Mr. Gladstone, concentrating perhaps on some morsel of Wedgewood china the great and complex engine of his mind; or on a later day Mr. Disraeli, fresh from private audience, and moved beyond his wont." Canon Duckworth, Dean Stanley, and Professor Tyndall were among the number of his instructors, and Sir Robert Collins, to whose guardianship he had early been committed, remained his best and life-long friend. Later on his entrance at Christ Church, Oxford, brought him many new friends among the leaders of the University as well as among the men of his own day. But probably no one exercised so powerful an influence on the moulding of his mind as Professor Ruskin. Mr. Collingwood's recent memorials of Ruskin have shown how dear to the master was his young Royal disciple. Mr. Myers' words must be quoted for the other aspect of the alliance: "There was one heart to which the Prince's heart went forth with a loving reverence such as he never felt for any other man. Certain colloquies of Mr. Ruskin's at the bedside of Prince Leopold, as he lay recovering from perilous illness, and still in danger of a relapse, will dwell in the mind of him who heard them as ideal examples of the contact of an elder and a younger soul. How close was that union in a region where earthly rank was swept away! How poor a thing did any life seem then which had not known the hallowing of sorrow! How solemn was that unspoken Presence which men have miscalled Death!"

From the time of his recovery from this dangerous attack of typhus fever the Prince's health slowly improved. After leaving the University and settling in his own country home of Boyton Manor in Wiltshire, he began to take some part in public matters, and was able to undertake travels on the Continent, extended in 1880 to Canada and the United States. In philanthropic projects of the most multifarious description he found his aid eagerly sought and his opinion courted. He shrank with real modesty from the position which many sought to thrust upon him of arbitration, or advocate among the conflicting efforts of the charitable, but his judgment once convinced, and his sympathies enlisted, he lent himself with full acceptance of responsibility to the cause he had chosen. The self-sacrifice and fatigues, seldom recognised by the public as inevitable to such work, were intensified in his case, and involved a severe strain on his powers of endurance, "though he met them with readiness, and would only jestingly allude to the inconvenience of holding one's hat three inches above one's head for a couple of hours in an east wind, or to the pains which he took to catch someone's eye in the crowd each time that he bowed and smiled, till his head grew too dizzy and his cheeks too strained for more than an automatic salute."

In speaking on behalf of the University College Hospital he says: "When men speak of Sir William Jenner, when they tell of his rare skill, his marvellous insight, his womanly tenderness, his unwearied care, then indeed can I not only endorse their sayings, but almost jealously assert that no one living has of their own knowledge and experience a better right to do so than myself, for more than once have they been put forth on my own behalf"; and later on he speaks of having "seen with our own eyes how the trials of sickness are aggravated by the absence of attention and comfort, which the rich indeed can purchase, but which the poor rarely find save within the kindly walls of a hospital."

There is but little more to tell of his life. In 1881, having been created Duke of Albany, Earl of Clarence and Baron Arklow, he made the acquaintance of Princess Helena of Waldeck Pyrmont, to whom the following year he was married in St. George's Chapel, Windsor. Two years of happy married life, passed for the most part in his new home at Claremont, and the end came. He died at Cannes of an attack of epilepsy induced by a fall, March 28th, 1884.

* "Science and a Future Life." By F. W. Myers. (Macmillan. 1893.)

